# Tackling Societal Challenges Related to Ageing and Transport Transition: An Introduction to Philosophical Principles of Causation Adapted to the Biopsychosocial Model

**DOI:** 10.3390/geriatrics1010003

**Published:** 2015-12-23

**Authors:** Paul Vaucher, Bernard Favrat

**Affiliations:** 1 School of Health Sciences Fribourg, University of Applied Sciences and Arts Western Switzerland, Route des Cliniques 15, Fribourg 1700, Switzerland; 2 Unit of Traffic Medicine and Psychology, University Centre of Legal Medicine, University Hospital of Lausanne (CHUV), Rue Saint-Martin 26, Lausanne 1005, Switzerland; 3 Department of Ambulatory Care and Community Medicine, University of Lausanne, Rue du Bugnon 44, Lausanne 1011, Switzerland; bernard.favrat@hospvd.ch

**Keywords:** traffic medicine, behavioural science, causality, transversal research

## Abstract

In geriatrics, driving cessation is addressed within the biopsychosocial model. This has broadened the scope of practitioners, not only in terms of assessing fitness to drive, but also by helping to maintain social engagements and provide support for transport transition. Causes can be addressed at different levels by adapting medication, improving physical health, modifying behaviour, adapting lifestyle, or bringing changes to the environment. This transdisciplinary approach requires an understanding of how different disciplines are linked to each other. This article reviews the philosophical principles of causality between fields and provides a framework for understanding causality within the biopsychosocial model. Understanding interlevel constraints should help practitioners overcome their differences, and favor transversal approaches to driving cessation.

## 1. Clinical Situation

A 78-year-old driver with a past history of cardiovascular disease was told his condition would not affect his fitness to drive, as long as he complied with his medication requirement to treat his arrhythmia. The patient is very attached to driving and attributes a feeling of freedom and independence to his car. He lives alone in a remote area and says he needs his car to do his shopping and meet with his friends and family. Recently, he has started suffering from mild cognitive impairment. A neuropsychologic assessment has nevertheless concluded that it was not yet severe enough to justify having to stop driving. It was therefore decided to monitor the situation and plan a transport transition phase within the next two years. He is now followed by an occupational therapist who has recently reported that the patient’s condition has worsened and that the patient expresses strong resistance to the idea of giving up driving. A meeting with the physician, the patient’s daughter, and the occupational therapist has been scheduled to discuss a solution to the problem and seriously consider driving cessation. Indeed, the patient’s mental health state had worsened to a point where he frequently forwent his treatment and occasionally drove with heart palpitations. After forgetting to take his medication several days in a row, his heart condition severely worsened while he was driving. He lost consciousness and his vehicle hit the side of the road before continuing onto the sidewalk. Unfortunately, at the same moment, a 14-year-old student, late for school, was speeding down the same sidewalk on his kick scooter and could not prevent himself from crashing into the side of the car, which had just come to a halt. The velocity of the impact immediately broke the young man’s left femur before he flew over the car and landed on the sidewalk, causing superficial skin wounds and a few bruises. What were the causes of the accident?

## 2. Introduction

Identifying the cause of such events has become important to define legal responsibilities, define modalities of reimbursements by insurance companies, improve health management, develop and promote evidence informed prevention policies, suggest changes in road regulations, and identify needs in infrastructures. For most drivers, the link between their age, their cognitive decline and their driving performance remains difficult to assess without an on-road evaluation [1,2]. There is indeed only a very weak association between reduced performance on neuropsychological tests and driving performance [3]. Furthermore, increased rates of traffic related deaths with age are mostly explained by decreased tolerance to injury rather than by age related changes in driving behaviour [4]. The physician’s role in assessing fitness to drive has therefore shifted from a pure traffic safety perspective [5,6] to a broader perspective that includes other priorities related to driving cessation [7], out-of-home mobility [8,9], mood disorders [10], and quality of life [11]. Physicians and medical practitioners are the privileged partners patients rely on to facilitate their transport transition and prevent social isolation and depression [12]. This biopsychosocial approach of driving cessation requires transdisciplinary skills and a better understanding of causality across disciplines. This article will therefore introduce philosophical principles of causation from different scientific domains and provide an ontological position adapted to the biopsychosocial model.

## 3. Ontological Positions

When working at the frontiers of different domains, it is very important to keep in mind that professionals do not necessarily have the same expectations from scientific work and do not necessarily rely on the same concepts to assert causal relationships. Taking a transversal approach towards a given problem therefore requires practitioners and researchers to recognise the role, strength and limitations of each domain and to clearly understand how they contribute to solving societal problems.

Basic science usually considers causality as the physical mechanism that explains the link between cause and effect. It aims for a better understanding of complex interactions in matter (mechanical approach). For epidemiologists, causality is seen as the link by which we can modify systems. This more practical approach, therefore, does not seek to explain causes but only to identify them (probabilistic approach). For example, in ageing populations, social engagement could prevent future disabilities [13]. In psychology, researchers mainly aim to model and explain behaviour through mental states using statistics (causal modelling). The Montreal Cognitive Assessment (MoCA) used to detect cognitive disorders is an example of causal modelling [14]. Sociologists mainly use statistics to test models associating social indicators to group behaviour (statistical inference). The emergence of geographical gerontology is a perfect example of the need to integrate notions from sociology into geriatrics [15]. Finally, causality in the legal system is founded in the concept of conformity. Moral entities are held responsible for an event if their behaviour has been non-conforming and it can be proven that the event was caused by this behaviour (non-conformity causality). Contraflow driving on the highway, for instance, would be considered as a cause of an accident, if it were to occur.

### 3.1. Mechanical Causality and Basic Science

Mechanical causality in science is the explanation of how parts of a system give certain properties to a whole system through the interactions between its parts [16]. Within a system, structural modifications of entities (effects) are produced by activities (causes) [17]. The physical exchanges that take place show regularity [18] and are therefore replicable in similar conditions (deterministic approach). It is therefore possible to study a one to one links between cause and effect. A condition becomes a cause when it inevitably leads to the effect and that this effect cannot occur without the condition [19]. This strict determinism approach is the milestone of basic science and is often referred to as sufficiency causation or necessity causation. In geriatrics, this approach of causality is widely used to identify and describe underlying biological mechanisms at a molecular level [17]. In our clinical scenario (see Section 1. Clinical situation), the fracture will depend on the propensity of the limb to deform and resist the applied force resulting from the impact of both moving objects. The mass of the car, intrinsic properties of bone to withstand loading and the trajectory of the kick scooter on the sidewalk are therefore some of the mechanical causes of the fracture. Identifying such causes can provide clues for preventive policies and also provide normative values for material safety regulations. Mechanical causality can also identify mechanism by which genetic factors can modulate cognitive decline and thereby define biomarkers indicating the onsets of a mental disease [20].

**Advantages**: In behavioural science, mechanical causality has many advantages over other definitions of causality. First, it can distinguish causes from consequences. Secondly, it explains underlying mechanisms that help consider possible solutions to prevent an event from taking place. Thirdly, the causal link is verifiable using an experimental approach in a controlled environment [21]. This approach is therefore epistemologically sound and provides a causal explanation we can rely on.

**Limitations**: Mechanical causality has some limitations. First, mechanical causality does not provide any information on the likelihood of a situation occurring and therefore cannot attribute different weight to different causes implied in a phenomenon. In other words, at a mechanical level, all causal factors are equally responsible for a given event. Secondly, defining mechanical causality highly depends upon the amount of control that can be exercised over the environment in which the studied phenomenon takes place. When studying complex phenomena, mechanical causality can therefore often arise in experimental or hypothetical observations that are not relevant in real life situations. Finally, mechanical causality is limited in determining states of complex uncontrolled systems through time. Laws that govern matter are not as deterministic as we presumed them to be. At a sub-molecular level, some forces that govern the organisation of matter are, by their nature, unpredictable [22]. These forces provide the scientific explanation of the part of unpredictability that can be observed at other levels (molecular, biochemical, cellular, intercellular, organism, inter-organism, environment etc.) and are called stochastic events. In our brains, stochastic events at a quantum level explain variations in gene transmission [23], in gene transcription [24], and in variability in synaptic transmission and plasticity [25,26]. Defining mental states as a mechanical cause of an accident is therefore close to impossible. Indeed, mechanical causality can only explain the state of brain activity for a few milliseconds.

### 3.2. Inter-Level Constraints

**Figure 1:**
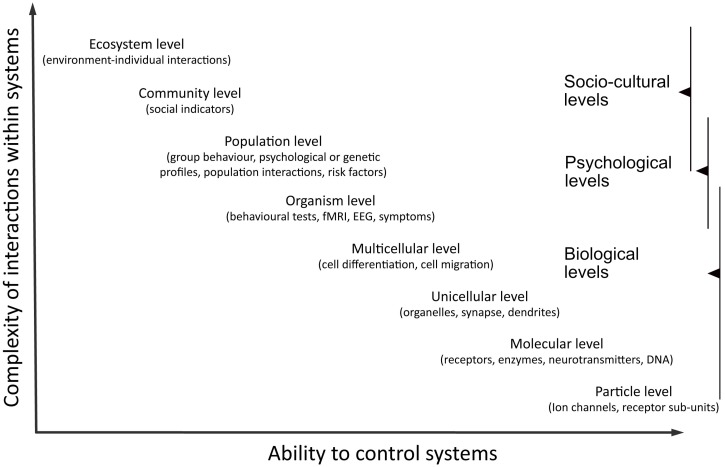
Trade-off between realism and control when working at different levels of organisation. At higher levels of complexity, causality is more difficult to assess. At higher levels of control, results are more difficult to apply in real-world situations.

Systems can be defined at different levels of organisation (Figure 1). If mechanical causation was all about reduction, then changes at any level should theoretically be explainable by interactions at any lower levels. This however does not apply to elementary particles. At this level, reduction becomes impossible. We can therefore admit that mechanical causality is designed to explain interactions between parts of a system but not necessarily to reduce the explanation to a lower level. Mechanical causation can even be used to explain consequences at a higher level. It is a common procedure to enhance changes at a molecular level by administrating or prescribing drugs, and then observe the effects on individuals’ behaviour (bottom-up experiments). An example is the prescription of anti-epileptic drugs and the absence of seizure during 12 months justifying fitness to drive [27].

We however also intervene at a behaviour level and then identify changes at a structural level (top-down experiments). Observing effects of a mindfulness based intervention on brain structural changes using MRI illustrates this [28]. Both these designs presumes we accept that mechanical causality can affect different levels of organisation. However, the direct link between effects at one level and consequences on another level is not straightforward. The epistemological grounding of inter-level causality becomes weaker and does not meet the criteria set by sufficiency causation or necessity causation. Kistler [16] overcame this problem by distinguishing constraints from causality. For him, "a constraint limits the possibilities of evolution or change accessible to a system" and this takes effect within and across levels. Therefore, mental causality can be seen as the result of constraints rising from neuronal activity [29]. Admitting inter-level constraints has major implications in the conception of mechanical causality in science. When studying mechanical causality across different levels, it is necessary to provide physical explanations of how changes at one level affect conditions at another level to the point that it constrains change in this system. In our fictional situation, inter-level constraints could provide explanations on why some patients with dementia deny encountering difficulties whereas others might tend to exaggerate them. The notion of inter-level constraints can also inspire grounded models on how to counter resistance to driving cessation and address the problem differently depending of the social context. Inter-level constraints also explains why we cannot certify that the accident would not have occurred had the driver taken his medication. Therefore, it remains impossible to know for certain if the driver’s cognitive impairment is one of the causes of the accident. This can be assumed uniquely if we base our inferences on a probabilistic approach.

**Advantages**: First, inter-level constraint widens the field of mechanical causality to include causality by omission and non-occurrence causality. The direct physical link between the absence of a cause and its effect does not have to exist any more. In other words, it is conceptually sound to consider that the absence of a cause at one level can modify constraints at another level. Public health is often concerned about how to prevent something from happening. When developing treatments, inter-level constraint is therefore indispensable to link the mechanical effect of the treatment with the non-occurrence of symptoms [30]. Secondly, inter-level constraints are essential for mechanical causality to provide explanations on how changes at a molecular level can enhance changes at a behavioural level and *vice versa*. This widens the field of application and supports treatments other than those at the molecular level. Finally, the concept of inter-level constraints opens the field of personalised medicine. Treatments can be adapted to individuals’ personal level of constraints.

**Limitations**: This approach nevertheless does not offer the possibility of accepting links between levels without acknowledging the physical constraints that one level has over another. Given the difficulty of providing such explanations at higher levels, we can use epidemiology, psychology and sociology to overcome this problem, by using a probabilistic approach to define causality. Epidemiology, psychology and sociology all rely on empirical observations and statistical inference to develop models of causation.

### 3.3. Epidemiology

Science might seek to know the extent to which conditions will tend to interact with each other and participate in an effect, without wanting to provide any explanation of underlying mechanisms. In this situation, a *probabilistic* approach is often preferred to a mechanical approach of causality. Even if mechanical causality can take multiple factors into consideration (sufficient-component causes) [31], this approach fails to take interactions into consideration or correctly model dose-effect response. Probabilistic causality in epidemiology has made it possible to attribute probability of multicausality, evaluate interactions among causes, and to estimate the strength of causes and the attributable fraction of a cause to a disease [32]. In observational studies, it is, however, often impossible to separate causal from non-causal associations. To limit false assumptions, Hill [33] proposed a list of nine viewpoints necessary to pass from the assumption of association to the one of causality (strength, consistency, specificity, temporality, biological gradient, plausibility, coherence, experimental evidence and analogy). There are, however, no absolute criteria for asserting the validity of scientific evidence in observational epidemiological studies. Therefore, Rothman [32] insists on the importance on relying on thorough criticism. Even experimental designs (randomised clinical trials) and meta-analysis are prone to bias that requires training and skills to recognise.

In our fictitious situation, probabilistic causality can estimate the attribution fraction of non-adherence to treatment in causing palpitations. It can also estimate the increased risk for road accident for drivers with different degrees of cognitive disorders [3], or predict the probability of being hit by a car when riding a kick scooter out on the street [34]. It can also predict the growing population of older drivers and help anticipate emerging problems [35].

**Advantages**: First, an epidemiological approach can construct models of interaction and take dose-response relations into consideration. These models can also easily takes stochastic events into consideration and can provide a long-term prediction of probability of future events. Secondly, probability causality can also attribute different weights to multiple causes and include factors from different levels of organisation (e.g., biologic markers, behaviour traits and social indicators). Finally, probabilistic causality can easily consider the absence of a cause, as a cause (counterfactuals) [36].

**Limitations**: The main limitation of epidemiology in science is that it does not provide any clear explanation of the underlying mechanisms linking a cause to an event. This approach is therefore entirely dependent on basic science and mechanical causality approaches to explain what it discovers. The second main difficulty is that probabilistic causality cannot distinguish simple associations from causal links unless we can assume that all other factors are held constant. Randomised clinical trials are therefore examples of controlled systems in which causal associations can be assumed. Conclusions can nevertheless subject to other sources of misinterpretation. Finally, for communication purposes, probabilistic causality in epidemiology most often concerns populations and not individuals. Patients often believe they are the exceptions and therefore do not always rely on conclusions drawn from populations. Focusing research on methods to improve clinicians’ communication skills for risks is often neglected in public health but should be set as a priority.

### 3.4. Psychology

Neuropsychological tests, behavioural questionnaires and psychological models are some of the instruments developed by causal modelling in psychology. In geriatrics, these instruments are used to assess cognition and behaviour by psychiatrists, psychologists, experts and primary care physicians [3,37]. They are developed after observing multiple patients with similar symptoms or unusual behaviour. From these observations, traits can be attributed as parts of a given mental state. Probabilistic causality is then used to assess the weight of each of these traits in defining the mental state. The final model’s ability to discriminate patient profiles with different mental states validates the entire procedure. This approach therefore helps provide reliable psychological explanations to certain mental states or behaviours.

For driving, psychological models can provide explanations on individuals’ acceptance of different level of risks, from which they adapt their driving strategy [38]. This influences their ability to anticipate, plan, decide and react to situations. When modelling driving cessation, we are however facing a major challenge; the difficulties in transposing results from neuropsychological tests to the real-life situation [39]. Geriatrics therefore needs to develop more complex psychological models that could serve to formulate guidelines for managing different profiles of patients who are resistant to transport transition. Psychology remains one of the most important contributors in traffic safety for finding adapted solutions to change people’s behaviour and prevent them from maintaining or adopting attitudes at risk [40].

**Advantages**: First of all, causal modelling makes it possible to quantify an individual’s mental state. Secondly, causal modelling also makes it possible to study interactions between different mental states in a single individual or between individuals. Finally, psychological causal modelling also provides precious indications of the risk of certain behaviours by individuals.

**Limitations**: This method assumes that subpopulations share common underlying mechanisms explaining their behaviour. These mechanisms however remain most often unknown. For similar mental states, different models can therefore come up with different explanations and classify conditions accordingly. This is done without been able to test the validity of a model over another. Relying on lower-level studies therefore remains essential to support causal modelling in psychology.

### 3.5. Social Science

Like for epidemiology and psychology, social science relies on statistical inference to model causal relationships. It often relies on global indicators and obtain the best "fit" between a constructed model and collected data [41]. Since the 80s, sociologists often rely on Mackie’s theory of causality: the INUS-condition (insufficient but necessary parts of a condition which is itself unnecessary but sufficient). This approach uses a set of conditions and guarantees non-redundancy. Approaches in sociology (INUS-condition, partial correlation and probabilistic cause) are based on correlation and association analysis. As in psychology, the concept of causality in social science is not as strong as in basic science. Sociology remains an interesting approach to identify associations between social indicators and societal events [41]. In geriatrics, sociology has largely contributed in understanding the meaning older drivers give to driving in their social context [42]. There is nevertheless a need for more to be done to account for social aspects of driving cessation when providing support to older drivers [43].

### 3.6. Non-Conformity Causality

There are multiple examples of social laws regulating our behaviour. Let us focus on road safety regulations. If the conditions necessary to cause an accident are in place, there is a given point in time when road users cannot change them and an accident becomes inevitable. Traffic regulations have therefore been defined to help road users anticipate events and act accordingly with each other, while preserving traffic fluidity as much as possible. In other words, road regulations are meant to give people sufficient time to identify danger, react in consequence and prevent collisions. These laws are governed by social agreement and conformity and as such are not deterministic. Nevertheless, not respecting such laws will modify constraints at a behavioural level. Road users will not be able to anticipate situations as expected. Non-conformity causality can thereby be linked to mechanical causality at a lower level. For non-conformity causality to take place, it remains essential to verify that in the given circumstances, had the person conformed to social rules, the event would not have taken place. Only under this condition should we consider non-conformity causality as a potential cause of events.

The usual way to consider non-conformity causality is to consider all the sufficient conditions for an event to take place, and then estimate which is unexpected or unusual and define it as the cause. In our fictive situation, driving a car on the sidewalk while unconscious and riding a kick scooter on the sidewalk do not conform to social expectations and can be considered as causes of the accident. If it can be proven that the young man would not have been injured had he been on foot, or that the senior driver would not have lost control of his car had he not forgotten to take his medication, both the young man and the driver could be held responsible for the accident. However, for someone’s responsibility to be engaged, it also has to be shown that they could have complied to social rules had they wanted to. In our clinical situation, the cognitive state of the driver could therefore disengage him from his responsibility.

**Advantages**: First, non-conformity causality can be studied by both probabilistic and mechanical approaches of causality. This approach can therefore provide explanations on the importance of societal rules in preventing or favouring events at a societal level. Secondly, this approach is the only approach that can attribute an individual’s level of responsibility to a given event. For this reason, it is often used by the legal system to establish links of causality between an individual’s action (or inaction) and damage.

**Limitations**: This type of cause is based on expectations and social conformity. The laws to which these causes respond are not absolute and can vary in time or space (different social groups can have different expectations and expectations can change over time). The constraint that this level has over other levels is therefore versatile, and transposing observations from one situation to another requires much caution.

## 4. Transdisciplinary approach

Human behaviour is influenced by many factors at many levels, making it one of the most complex systems to study in science. As we have seen, defining unfitness to drive can range from identifying irreversible molecular changes in neural networks to identifying an individual’s incapacity to adapt their driving behaviour to new road regulations. With cognitive decline, the problem of driving cessation and traffic safety has been shown to be much less important as initially presumed except for very advanced conditions that also makes other daily tasks at home difficult [3,44,45]. The health impact of forced driving cessation seems to be of higher concern [7,46]. There is therefore a natural shift in the domain towards also addressing the problem of social isolation and independence rather than traffic safety alone [12]. This shift is inevitable given cars are already equipped with automatic braking systems, parking systems, and new technology increasing passengers’ security. In a close future, it will be more common that cars are programmed to anticipate events and automatically prevent accidents. Therefore, we might encounter potential problems concerning older drivers who want to maintain control over their vehicle. Traffic safety research also needs to tackle the question of how to help older drivers trust emerging technologies. This requires psychologists, engineers, geriatrics, neuroscientists to work together across different levels of organisation [47]. Higher-level research provides important clues on which hypothesis to test at lower levels. Higher-level research can also provide feedback on the applicability of lower-level discoveries and help decide when to abandon or favour a lead. On the other hand, lower-level research provides explanations for higher-level interactions. This can help improve systems to target goals more efficiently and improve predictions. Transversal research therefore embraces problems in their complexity and provides coordinated interpretations of results across multiple levels to explore, develop, and assess possible applicable solutions to a problem [48]. These efforts can be enhanced by prompting interdisciplinary collaborative projects. Limitations from one domain can be compensated by advantages from another; there is a trade-off between fidelity and experimental control (Figure 1). For clinicians and researchers from different fields to work together, it helps to keep in mind that accepting a causal link is highly dependent of the compatibility of any proposition with their own belief system [49]. We will therefore probably remain suspicious of other sources of knowledge than our own. This scepticism is beneficial as long as we put efforts in continuously aligning our concepts of causality to the ones of other speakers. Building a consortium of clinicians or researchers who can focus on common goals within a clear framework is probably the most cost-effective way of finding optimal adapted solutions to transportation needs and safety.

## 5. Conclusions

In respect of the biopsychosocial model, the psychological and social aspect of driving cessation need to be accounted for when managing older patients with emerging cognitive deficits. Helping older patients through this transition phase is a way of preventing the burden of social isolation [50]. Research has however neglected this aspect of driving cessation; maybe due to the difficulty of addressing age related needs in transport transition by a single domain. There is an urgent need for updated guidelines that take all dimensions of driving cessation into account. Collaborative transdisciplinary approaches could help find optimal implementable solutions to help clinicians address the problem. In complex uncontrolled systems, conditions vary considerably between individuals and within individuals at different time frames. Therefore, causes at one level can have a wide variability of effects at another level. It is only through inter-level constraints that modifications at one level can alter conditions at another level. This concept of inter-level constraints provides a solid foundation for clinicians and scientists from different domains to work with one another and highlights the importance of each discipline in achieving common goals.
